# Upon the Difference of Temperature between Venous and Arterial Blood

**Published:** 1853-11

**Authors:** G. V. Liebig


### ANATOMY AND PHYSIOLOGY.

*Upon the difference of Temperature between Venous and Arterial Blood.* By G. V. Liebig.—Former investigations upon this subject have led to conflicting results. Most of the older observers, such as Haller, Crawford, Kramer, Scudamore, &c., having declared, that the arterial blood (in the left cavities of the heart) is warmer than the venous blood of the right cavities by 1°; while A. Cooper, Coleman, Mayer, Autenrieth, etc., either pronounce the temperature of both kinds of blood equal, or give to the venous blood an excess of temperature over the arterial of 0·5°. The author proceeds, in his attempts to explain these differences, to inquire, whether the higher temperature be found in the blood flowing from the lungs, or in that from the capillaries, and he reverts to some of his former observations upon "muscular respiration."

The experiments were conducted both upon living and recently-killed animals. That death might be caused quickly, and without loss of blood, the medulla oblongata was severed in some instances, and narcotin was administered in others. The thermometer was introduced, either by the jugular vein or the carotid artery, to the requisite depth of the heart; or it was inserted into the crural vein, or the vena cava abdominalis. Respiration was prolonged after death, when necessary. The experiments were performed upon dogs, and with every possible precaution to avoid errors,

The results were—1. That the temperature of the blood in the right cavities of the heart exceeds that of the left cavities by 0·05°; 2. That the blood flowing into the heart by the vena cava descendens is cooler than that from the vena cava ascendens (probably because the blood which the former vessel contains comes from parts which presents to the atmosphere a very great extent of surface in which a process of cooling takes place.)

In the venous system, there go on changes of temperature corresponding with the respiratory act. In the vena cava superior it was remarked that at the end of each inspiration the temperature of the blood rose; between inspiration and expiration it attained its maximum; towards the end of expiration it fell; and was at its lowest point after expiration. When animals breathe, or rather expire, shortly and quickly, and then inspire deeply, the variations of temperature are greater than usual. When the thermometer is in the right auricle, the highest degree corresponds with inspiration, and the lowest with the beginning of expiration. When the breathing is very short and hurried, these changes of temperature become diminished, or even cease altogether. In the vena cava abdominalis the same phenomena were not observed; in the vena iliaca they were reversed, the maximum occurring after expiration, and the minimum after inspiration.

The author then remarks upon the mechanical influence exerted by the respiratory movements upon the circulation in veins. The enlargement of the thoracic, and the diminution of the abdominal cavities, coincident with inspiration, cause the blood of the vena cava ascendens to flow into the right auricle, during and especially at the end of inspiration. The contents of the vena cava descendens are less than those of the vena cava ascendens, and they are cleared out at the beginning of inspiration. During expiration the abdominal cavity is widened, and the vena cava abdominalis gains both in space and contents. Towards the end of inspiration, the auricle will therefore be filled by the warm blood of the vena cava abdominalis; and at this moment is observed the highest degree of variation in the heart's temperature. In expiration the blood streams into the auricle chiefly from the vena cava descendens, and then is remarked the minimum of temperature. As regards differences of temperature in different parts of the same system, the author found the blood of the vena cava sup. 0.16°, and that of the auricle 0.20°. Between the auricle, where the blood from two vessels of different temperature is not completely mixed, and the ventricle, the difference amounted to 0.1—30.20°.

In the arterial system there are no variations dependent upon the movements of respiration, or they are very slight, amounting to 0·01—0.06°, and connected with the changes in the right cavities of the heart, and the cooling of the blood in its passage from the lungs.

In dead animals, the temperature of the blood of the right ventricle exceeds that of the left by 0·16°. In two experiments, however, the temperature was found the same in both cavities. The temperature of the blood of the vena cava abdominalis was once, upon opening the chest, found 1·72° higher than that of the thoracic portion of the carotid during life. The same observations were repeated in several experiments. The blood of the vena cava abdominalis, which comes from the capillaries of the lower extremities, and of some of the abdominal viscera, is the warmest blood in the body, even in animals where the heart has been emptied by bleeding, and to this point the author attaches great importance.

It would be desirable to know the amount of cooling which the blood undergoes in its passage through the lungs; but the subject is fraught with difficulty. The fact, however, if established, is most important, inasmuch as the source of animal heat has been referred by many to the organs of respiration. As regards the warmth of the blood in the vena cava abdominalis, it must be remembered, that the vessel lies imbedded in soft, highly organised parts, where, under all circumstances, heat is retained for a longer time than in other parts of the body; and perhaps this fact will explain the cause of the warmth of blood in that vessel as satisfactorily as any theory of changes going on in the capillary system. —*Med. Times, from Schmidt's Jahrb.*, 1853.

